# Supplementary material for: Complexome profiling of the Chlamydomonas psb28 mutant reveals TEF5 as an early PSII assembly factor
Source: Plant Cell. 2025 Mar 17;37(6):koaf055. doi: 10.1093/plcell/koaf055 (PMC12164590; doi:10.1093/plcell/koaf055)
Supplement: koaf055_Supplementary_Data [file koaf055_supplementary_data.zip › psb28_interactive.zip!/plots/intensity/qualityPlots/peptides/TEF11_Cre03.g145247.t1.1.html]

### Cre03.g145247.t1.1

Trivial name: TEF11  
SeqCoverage [median]: 35.90  
Mass: 24.02 kDa  
  
 

| Sequence | MissedCleavage | Mass [Da] | wtIntensity | muIntensity | Score |
| --- | --- | --- | --- | --- | --- |
| AKSGASTDADMFASK | 1 | 1485.68 | 0.00 | 632.70 | 93.42 |
| ALEELYPEYPQYK | 0 | 1641.79 | 17432.29 | 52974.42 | 222.30 |
| ANLEEK | 0 | 702.35 | 1950.29 | 6971.56 | 157.99 |
| ATDDASDPVPK | 0 | 1114.51 | 16397.00 | 33312.21 | 166.34 |
| ATDDASDPVPKAK | 1 | 1313.65 | 0.00 | 1815.29 | 135.02 |
| KFIPYLL | 1 | 892.54 | 467.39 | 0.00 | 74.60 |
| KNHALVTDGLYALSR | 1 | 1656.89 | 0.00 | 10900.21 | 106.30 |
| NHALVTDGLYALSR | 0 | 1528.80 | 0.00 | 2224.21 | 55.88 |
| QLSLSPAFSVAPK | 0 | 1343.74 | 0.00 | 50167.68 | 69.28 |
| SGASTDADMFASK | 0 | 1286.54 | 5906.05 | 18033.89 | 179.19 |
|  |  |  | 42153.0 | 177032.2 |  |
